# Efficacy and Safety of Nanoparticle Albumin-Bound Paclitaxel in Elderly Patients with Metastatic Breast Cancer: A Meta-Analysis

**DOI:** 10.3390/jcm8101689

**Published:** 2019-10-15

**Authors:** Xin Li, Hyungju Kwon

**Affiliations:** Department of Surgery, Ewha Womans University Medical Center, 1071 Anyangcheon-ro, Yangcheon-Gu, Seoul 07985, Korea; yikicat@naver.com

**Keywords:** paclitaxel, metastatic breast cancer, nanoparticle albumin-bound

## Abstract

Nanoparticle albumin-bound paclitaxel (nab-paclitaxel) is an approved treatment for metastatic breast cancer (MBC). However, there is an ongoing debate about the efficacy and safety of nab-paclitaxel in elderly patients. We conducted a meta-analysis to evaluate nab-paclitaxel efficacy and adverse events in MBC patients 65 years and older, compared with MBC patients younger than 65 years (control group). We performed a literature search using PubMed, the Cochrane Library, and EMBASE, from their inception to 30 September 2019. The relevant studies compared overall response rates (ORRs) and incidence of adverse events; four studies comprising 1204 patients were identified and included. ORRs were similar in patients older than 65 years and controls (odds ratio (OR) 0.71, 95% confidence interval (CI) 0.42–1.21). On subgroup analysis, both first-line therapy (OR 2.54, 95% CI 1.92–3.36) and lower Eastern Cooperative Oncology Group (ECOG) performance status (OR 0.20, 95% CI 0.06–0.69) were associated with a higher ORR. Adverse events including neutropenia, sensory neuropathy, diarrhea, and nausea were comparable between the groups. In conclusion, nab-paclitaxel showed comparable efficacy and safety in older and younger patients with MBC. Nab-paclitaxel can be a first-line treatment option for MBC patients 65 years and older.

## 1. Introduction

Breast cancer is the most common malignancy and the leading cause of cancer death in women [1]. In 2018, there were approximately 2 million patients with newly diagnosed breast cancer, accounting for 11.6% of all cancer cases worldwide [1]. Ladoire et al. showed that nearly a third of breast cancers occur in patients older than 65 years, and this proportion reaches more than 40% in developed countries [2]. Although chemotherapy is usually recommended for women with a high recurrence risk of breast cancer, older women may be less likely to receive chemotherapy than younger women [3,4]. As patients over 65 are likely to metabolize chemotherapeutic agents more slowly or differently than younger patients, higher levels of drug exposure may occur and result in more serious adverse events [5,6].

Cytotoxic chemotherapy is commonly administered to patients with metastatic breast cancer (MBC). Paclitaxel is among the most widely used chemotherapeutic agents for breast cancer; hypersensitivity reaction is one of its major drawbacks [7]. To decrease the risk of hypersensitivity, an alternative nanoparticle albumin-bound paclitaxel (nab-paclitaxel, also known as Abraxane^®^) was developed [6]. As nab-paclitaxel eliminates the need for toxic solvents, such as Cremophor, it affords more flexible dosage and overall improved efficacy [8]. Nab-paclitaxel has many demonstrated advantages, such as less need for premedication, a shorter infusion time, and a higher response rate, compared to standard paclitaxel. However, there is an ongoing debate about the associated toxicities, including sensory neuropathy, nausea, and diarrhea [9,10,11]. Further assessment of these effects in elderly patients, who may be more vulnerable to the chemotherapeutic agent-associated toxicity, is needed. Therefore, we conducted a meta-analysis of nab-paclitaxel efficacy and toxicity in MBC patients 65 years and older, compared with younger patients.

## 2. Materials and Methods

### 2.1. Literature Search Strategy

This meta-analysis was conducted following the recommendations of the Preferred Reporting Items for Systematic Reviews and Meta-Analyses (PRISMA) [12]. The following databases were searched from inception to 30 September 2019: PubMed/MEDLINE (*n* = 454), EMBASE (*n* = 993), SCOPUS (*n* = 975), the Cochrane Database of Systematic Reviews (*n* = 4), and Web of Science Core Collection (*n* = 984). Two authors (X.L. and H.K.) independently performed the review with the search terms (“albumin-bound paclitaxel” or “nab-paclitaxel” or “abraxane”) and (“breast”) using the Boolean ‘AND’ operator.

### 2.2. Eligibility Criteria

The inclusion criteria were as follows: (1) studies that involved patients with metastatic breast cancer receiving nab-paclitaxel-based chemotherapy, (2) studies that included statistical data on younger and older patients, (3) outcomes measured: therapeutic response of tumor and/or adverse events, and (4) no limitation for study design.

**Figure 1:**
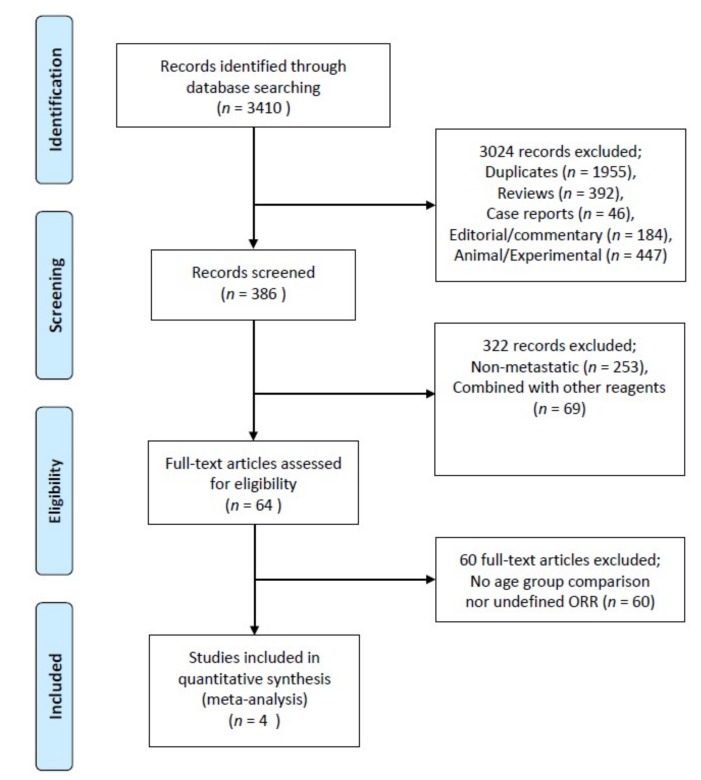
The Preferred Reporting Items for Systematic Reviews and Meta-Analyses (PRISMA) flow diagram of the included studies.

The exclusion criteria were as follows: (1) studies on patients with stage I–III breast cancer, (2) case reports, editorials, and commentaries, (3) nonhuman studies including animal studies and experimental studies, and (4) articles not written in English (Figure 1).

### 2.3. Data Extraction and Quality Assessment

Data including names of authors, publication year, study design, patient age, breast cancer hormone receptor status, treatment schedule, therapy regimen including dosage, overall response rate (ORR), and rates of adverse events (only grade 3 to 4 toxicities) were gathered with a structured data collecting form. We applied the Newcastle–Ottawa scale for assessment of study quality [13]; only studies with scores of 7 or higher were eligible for inclusion in this meta-analysis.

### 2.4. Statistical Analysis

Review Manager Version 5.3 (Cochrane Collaboration, Oxford, UK) was used to conduct all the statistical calculations. Odds ratio (OR) and corresponding 95% confidence intervals (CIs) were used to compare the outcomes; namely, ORRs and adverse events. Statistical heterogeneity among these studies was calculated by Cochran’s Q test and the I^2^ index (≤25% = insignificant heterogeneity, 26–50% = low heterogeneity, 51–75% = moderate heterogeneity, and over 75% = high heterogeneity) [14]. The random-effects model was used when moderate or greater heterogeneity was present among studies; otherwise, the fixed-effects model was applied. Publication bias was evaluated by funnel plots [15].

## 3. Results

### 3.1. Search Results

A total of 3410 potentially eligible records were identified in the electronic databases. After excluding 3024 articles that were duplicates (*n* = 1955), reviews (*n* = 392), case reports (*n* = 46), editorial or commentaries (*n* = 184), and experimental studies (*n* = 447), we selected 386 articles for possible inclusion. Of these, 322 articles that did not focus on MBC treatment (*n* = 253) or involved the use of other reagents (*n* = 69) were subsequently excluded. We performed a full-text review of the remained 64 articles; 60 of those were additionally excluded because they did not report ORRs or did not compare age groups. Finally, four articles including a total of 1204 patients were selected. The search results and characteristics of the included studies are described in Table 1.

### 3.2. Overall Tumor Response Rates

**Figure 2:**
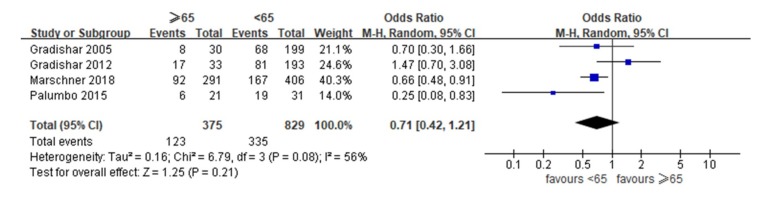
Forest plot of the included studies assessing differences in overall response rates between patients ≥65 years and patients under 65 years.

Figure 2 shows the meta-analysis findings. The ORRs ranged from 26.6% to 51.5% in patients 65 years and older and from 34.2% to 61.3% in patients <65 years (control), respectively. As moderate heterogeneity was observed across studies (I^2^ = 56%, *p* = 0.21), we used random-effects models. Compared to the control patients, the patients 65 years and older had similar ORRs (OR 0.71, 95% CI 0.42 to 1.21, *p* = 0.08).

### 3.3. Subgroup Analysis for Prediction of Responses

We analyzed six factors that might affect the ORR: ECOG performance status (0 or ≥1), receptor status (non-TNBC or TNBC), pretreatment (taxane pretreatment or no pretreatment), treatment line (first-line or higher), treatment schedule (weekly or triweekly), and dominant metastatic sites (visceral or nonvisceral). Detailed outcomes of the possible predictive factors are described in Table 2. In the subgroup analysis, both ECOG performance status and treatment line showed statistical significance. An ECOG score of 0 was associated with a higher ORR than an ECOG score ≥1 (OR 0.20, 95% CI 0.06 to 0.69, *p* = 0.01). First-line treatment also increased the ORR to nab-paclitaxel compared with higher line treatment (OR 2.54, 95% CI 1.92 to 3.36, *p* < 0.001).

### 3.4. Adverse Events

**Figure 3:**
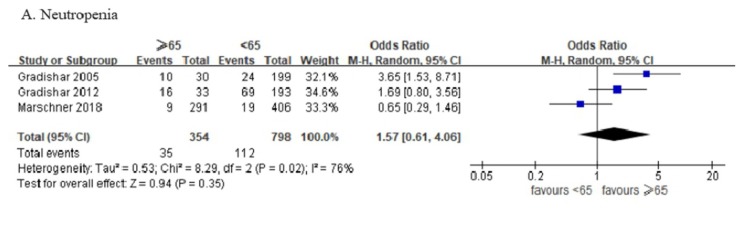

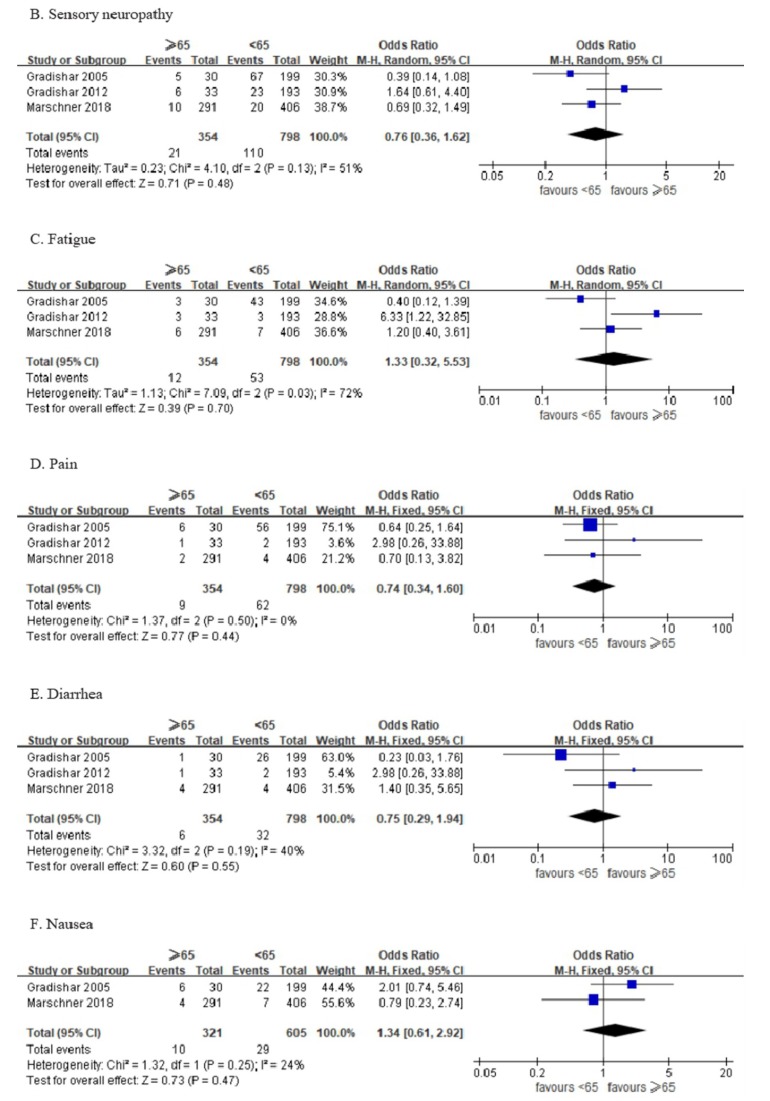
Forest plot of the included studies assessing (**A**) neutropenia, (**B**) sensory neuropathy, (**C**) fatigue, (**D**) pain, (**E**) diarrhea, and (**F**) nausea between patients ≥ 65 years and patients under 65 years.

Three articles including 1152 patients provided complete toxicity profiles; one article did include data on nausea events. All adverse events higher than grade 3 were identified and analyzed. There were no significant between-group differences for neutropenia, sensory neuropathy, fatigue, pain, diarrhea, and nausea (Figure 3).

### 3.5. Publication Bias

**Figure 4:**
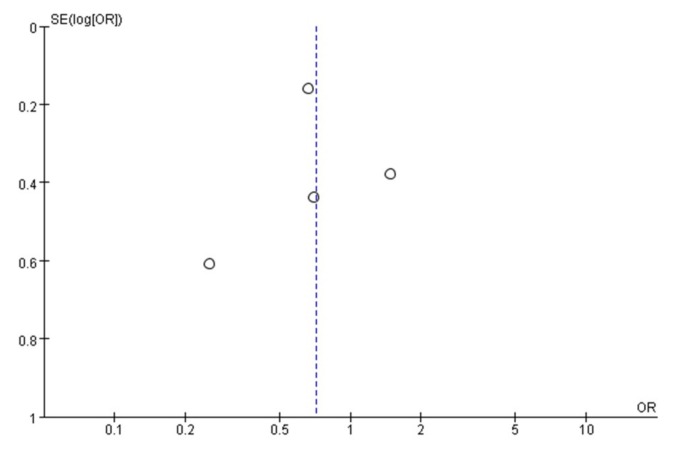
Funnel plot demonstrating the absence of publication bias in the response rate analyses.

A funnel plot was used to assess publication bias in the studies evaluating overall response rates to nab-paclitaxel. No publication bias was detected (Figure 4).

## 4. Discussion

This meta-analysis demonstrates similar efficacy and toxicity of nab-paclitaxel in patients 65 years and older with MBC, compared with those younger than 65 years. Because of the aging of the population and the steep rise of cancer incidence with age, the prevalence of cancer in the population aged 65 years and older is expected to increase by approximately 70% between 2010 and 2030 [19]. With the increased numbers of elderly patients with breast cancer, the efficacy and safety of chemotherapy in this population is a major consideration. The National Comprehensive Cancer Network guidelines for breast cancer state that the selection from among the various local or systemic therapies should be based on several factors including patient age [20]; however, the panel concluded that there were insufficient data to make definitive recommendations for chemotherapy in women older than 70 years.

Taxanes, including paclitaxel, are considered the most effective cytotoxic drugs for the treatment of MBC, both in monotherapy and combination schedules, with a proven survival benefit compared with other types of chemotherapy [6]. Recent studies showed that nab-paclitaxel also had excellent efficacy in either single or combined treatments for MBC patients [21,22,23]. In randomized trials, compared to standard paclitaxel, nab-paclitaxel has shown at least comparable or higher response rates with lower toxicity [9,10]. However, the efficacy and safety of nab-paclitaxel were not fully established in patients over 65 years. We performed the meta-analysis to address this unmet need.

Efficacy analysis of the 4 qualified studies showed a comparable response to nab-paclitaxel in patients 65 years and older and controls. Both first-line therapy (OR 2.54, 95% CI 1.92–3.36) and lower ECOG performance status (OR 0.20, 95% CI 0.06–0.69) were significantly related to higher response rates, regardless of patient age. Other factors including administration schedule, receptor status, and dominant metastatic sites were not associated with the efficacy of nab-paclitaxel. These results indicate that nab-paclitaxel could be a good choice for first-line treatment of MBC in elderly patients with lower ECOG scores. Although some investigators have suggested that a weekly treatment schedule results in better response rates in these older patients than once per three weeks dosing, our results showed no difference (OR 1.30, 95% CI 0.92–1.85; *p* = 0.14) [5].

The major adverse effects of paclitaxel include myelosuppression, alopecia, musculoskeletal discomfort, and hypersensitivity reactions [24]. Compared to standard paclitaxel, nab-paclitaxel has shown higher rates of some adverse events, including sensory neuropathy (71% vs. 56%), fatigue (47% vs. 38%), arthralgia (35% vs. 33%), nausea (30% vs. 21%), infections (24% vs. 20%), and diarrhea (26% vs. 15%), whereas the rates of alopecia (90% vs. 94%), neutropenia (34% vs. 49%), and myalgia (28% vs. 32%) were lower [25]. In our analysis of the qualified studies, we observed comparable adverse event rates in the older patient and control groups, indicating that nab-paclitaxel can be used in elderly patients with MBC without additional risk of adverse events.

This meta-analysis has some limitations. First, only four articles focused on chemotherapy in older patients with MBC were identified. The relatively small number of patients could have resulted in multiple types of bias. Second, a previous study indicated that a nab-paclitaxel dose of 125 mg/m^2^ was associated with a better safety profile and compliance without compromising efficacy, compared to 150 mg/m^2^ [26]. However, we were unable to consider the dosage of nab-paclitaxel in the present meta-analysis because of the limited number of qualified studies. Third, we did not evaluate long-term outcomes and late complications. Further studies are needed to draw more precise conclusions.

## 5. Conclusions

Nab-paclitaxel showed comparable efficacy and safety in older and younger patients with MBC. Both first-line therapy and lower ECOG performance status predicted higher response rates. In conclusion, nab-paclitaxel can be a first-line treatment option for MBC patients ≥65 years.

## Figures and Tables

**Table 1:** Characteristics of the included studies.

Study	Year	Design	Number of Patients		Receptor Status	Treatment Line	Responder (%)		NOS
			≥65 Years	<65 Years			≥65 Years	<65 Years	
Palumbo [16]	2015	Prospective	21	31	HER2(-) only	Second line	6 (28.6)	19 (61.3)	9
Marschner [17]	2018	Prospective	291	406	All	First or higher line	92 (31.6)	167 (41.1)	8
Gradishar [10]	2005	Prospective	30	199	NR	NR	8 (26.6)	68 (34.2)	8
Gradishar [18]	2012	Prospective	33	193	NR	First line	17 (51.5)	81 (41.9)	7

Abbreviations: NOS, Newcastle–Ottawa scale; HER2, epidermal growth factor receptor 2; NR, not recorded.

**Table 2:** Predictive factors for overall response rate to nab-paclitaxel.

Predictive Factors	Subgroup (Responders/Total Patients)		OR (95% CI)	I^2^ (*p*-Value)
ECOG performance	0 (20/32)	≥1 (5/20)	0.20 (0.06–0.69)	NA
Receptor status	Non-TNBC (207/539)	TNBC (45/112)	0.65 (0.17–2.43)	75 (0.04)
Taxane pretreatment	Yes (162/435)	No (122/304)	0.93 (0.68–1.25)	17 (0.27)
Treatment line	First (170/377)	Higher (134/549)	2.54 (1.92–3.36)	0 (0.36)
Treatment schedule	Weekly (192/491)	Triweekly (64/194)	1.30 (0.92–1.85)	NA
Metastatic sites	Visceral (81/211)	Nonvisceral (20/67)	2.52 (0.33–19.5)	85 (0.009)

Abbreviations: OR, odds ratio; CI, confidence interval; ECOG, Eastern Cooperative Oncology Group; NA, not applicable.
